# High-predation habitats affect the social dynamics of collective exploration in a shoaling fish

**DOI:** 10.1126/sciadv.1602682

**Published:** 2017-05-03

**Authors:** Christos C. Ioannou, Indar W. Ramnarine, Colin J. Torney

**Affiliations:** 1 School of Biological Sciences, University of Bristol, Bristol, U.K.; 2 Department of Life Sciences, University of the West Indies at St. Augustine, St. Augustine, Trinidad and Tobago.; 3 School of Mathematics and Statistics, University of Glasgow, Glasgow G12 8QW, U.K.

**Keywords:** Group decision making, predation, self-organisation, collective behaviour, cross-population, leadership, guppy, initiators, followers

## Abstract

Collective decisions play a major role in the benefits that animals gain from living in groups. Although the mechanisms of how groups collectively make decisions have been extensively researched, the response of within-group dynamics to ecological conditions is virtually unknown, despite adaptation to the environment being a cornerstone in biology. We investigate how within-group interactions during exploration of a novel environment are shaped by predation, a major influence on the behavior of prey species. We tested guppies (*Poecilia reticulata*) from rivers varying in predation risk under controlled laboratory conditions and find the first evidence of differences in group interactions between animals adapted to different levels of predation. Fish from high-predation habitats showed the strongest negative relationship between initiating movements and following others, which resulted in less variability in the total number of movements made between individuals. This relationship between initiating movements and following others was associated with differentiation into initiators and followers, which was only observed in fish from high-predation rivers. The differentiation occurred rapidly, as trials lasted 5 min, and was related to shoal cohesion, where more diverse groups from high-predation habitats were more cohesive. Our results show that even within a single species over a small geographical range, decision-making in a social context can vary with local ecological factors.

## Teaser

Comparing across populations, we show that predation has multiple effects on collective exploration in groups of guppies.

## INTRODUCTION

The risk of predation is a major force in the evolution of morphological, behavioral, and life history traits (*1*). Stunning examples of collective movement, such as the dynamics of fish schools (*2*) and bird flocks (*3*), are widely believed to be adaptations to avoid predation (*4*–*6*). Despite this, there is little understanding of the effect of predation on another major aspect of living in groups—how animals make decisions in a social context. In a wide range of situations and species, group decision-making improves the ability to search for, detect, and appropriately respond to predators and resources (*7*–*9*). A great deal of attention has been given to the mechanism(s) by which groups collectively make decisions, for example, the use of quorum decision-making rules and how this may be adaptive (*10*–*12*). Other works have focused on whether group decisions are equally shared (that is, egalitarian) or decided by a minority of the group, as occurs during leadership (*13*–*15*), often investigating how traits of group members are associated with influence on group decisions (*16*–*19*). Most of this research has studied single populations of a species, and whether the social interactions that underpin group decisions can respond to ecological factors remains to be investigated. Prey species from higher-predation habitats have already been shown to form larger and more cohesive groups (*5*, *20*, *21*), suggesting the rules of how individuals respond to each other when interacting socially are shaped by predation pressure. Although predation is also known to affect individual-level behavior that is relevant to influencing group decisions, such as exploration (*22*) and learning (*23*), how social interactions in the context of decision-making vary with predation pressure is unknown.

Leadership in animal groups, where some individuals have a disproportionately large effect on the decisions made by a group, occurs through a number of different mechanisms. In groups such as fish shoals, information transfer is believed to occur through changes in movement rather than active signaling (*24*) and generally requires leaders to take positions at the front of the group (*25*). Through a passive process, individuals may have a greater influence on the direction of moving groups by being at the front (*26*). Alternatively, particular individuals may have a greater tendency to initiate movements or attempt to change the group’s direction of travel due to a greater metabolic need (*19*, *27*), reduced perception of risk (*28*, *29*), or because they have information (for example, regarding the location of food) not held by others in the group (*30*). On the basis of previous work, predicting the effect of predation on tendencies to initiate movements or follow others is not straightforward. On the one hand, being at the front of groups often increases encounter rates with predators (*31*); thus, higher predation risk may select for more egalitarian groups because of individuals reducing their tendencies to lead, resulting in greater conformity and more cohesive groups (*29*, *30*). In contrast, if tendencies to initiate movements are a result of a trade-off between predation risk and the rate of acquiring food, which also tends to be greater for individuals at the front of groups (*27*), increased predation risk may act to induce a stronger trade-off and magnify differences between individuals. This may explain why predation pressure has been associated with an increased consistency of interindividual variation in behavior (*32*), and if these behavioral traits are associated with individual tendencies to lead (*18*, *33*), this population-level variation may cause groups to be less egalitarian. Interactions within groups can also result in differentiation into leader and follower roles, an effect referred to as social feedback (*28*), where individuals change their behavior to be different from their group mates.

Here, we aim to determine how collective exploration of novel environments is shaped by local selective pressure driven by predation, using Trinidadian guppies. Guppies in the Northern Range mountains of Trinidad live in geographically isolated rivers; in many of these rivers, dispersal of predators upstream is limited by waterfalls and rapids, so guppy populations in lowland reaches coexist and evolve under a substantial threat of predation, whereas in upland reaches, the risk of predation is substantially reduced (*34*, *35*). Trinidadian guppies have become a well-established model system for exploring the role of predation in the evolution of vertebrate behavior, life history, and morphology using a cross-population comparative approach (*1*). Guppies from high-predation habitats have been shown to have increased shoaling (*20*, *21*, *36*, *37*) and risk-taking behavioral tendencies (*38*), both of which can affect decisions in groups.

We compared fish caught from sites varying in predation risk and tested the tendencies of individual fish in groups to initiate movements and to follow others, as well as the relationship of this with group cohesion. Predation is expected not only to reduce exploration (*39*), resulting in fewer initiations, but also to increase group cohesion (*20*, *21*, *36*, *37*) and the tendency to follow. As individuals from higher-predation habitats have been shown to be more consistent in their behavior across contexts (*40*), we predict that there may be greater variation in tendencies to lead and follow between individuals in fish from high-predation habitats. If individuals within groups change their behavior based on that of other fish in the group [that is, via social feedback (*28*)], this may be increased by the greater social tendencies of fish from high-predation habitats (because more social individuals may be more responsive to the behavior of others) or reduced due to a greater conformity effect in more social fish. Group cohesion may thus be positively or negatively associated with the variation within groups in the tendency to initiate movements if within-group variation is affected by social feedback.

## RESULTS

**Fig. 1:**
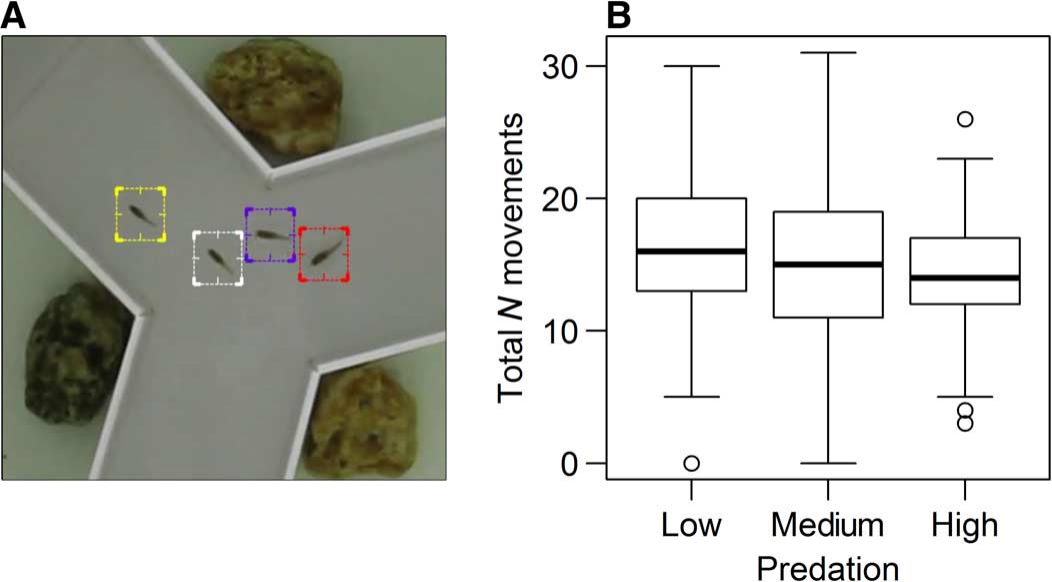
Experimental design and number of movements between the arms of the maze. (**A**) A zoomed-in still from movie S1 of a fish (yellow) initiating a movement into an empty arm and leading the three other fish. (**B**) The effect of predation risk in the source habitat on the total number of movements between arms per individual fish. The median is shown by the solid line. The interquartile range is enclosed by the box. The whiskers extend to the most extreme data point within 1.5× the interquartile range outside the box, and empty circles show data points beyond the range of the whiskers. Predation in the source habitat had no effect on average levels of activity [linear mixed model (LMM): *F* = 0.27, *P* = 0.61], but individuals were less variable when they were from high-predation habitats [negative binomial generalized linear mixed model (GLMM) on model residuals: χ^2^ = 6.70, *P* = 0.0098]. This reduced variability in fish from high-predation rivers was confirmed with Levene’s test (*F*_2,307_ = 9.68, *P* = 8.41 × 10^−5^).

We used two populations of guppies from each of the three rivers listed in table S1 and broadly classified the predation risk of each population based on predator species at the collection sites as follows: high (an abundance of *Crenicichla frenata*, a major predator of adult guppies), medium (absence of *C. frenata* but presence of *Hoplias malabaricus* and *Aequidens pulcher*, opportunistic and occasional guppy predators), and low (presence of only *Rivulus hartii*, a minor predator of juvenile guppies). We tested single-sex groups (that is, all male or all female) under controlled laboratory conditions in a radially symmetric three-armed maze and recorded their movements for 5 min using software that maintained individual identities (Fig. 1A and movie S1). Guppies show highly dynamic fission-fusion social behavior, where individuals exchange between groups over the scale of minutes (*41*); thus, 5 min is an ecologically relevant time period to study group behavior in this species. Groups of two and four individuals were tested; pairs are commonly used in studies of collective behavior in fish (*18*, *28*, *33*), and testing larger groups of four individuals allowed us to examine whether any effects of predation pressure in pairs generalized to, or differed from, the effects of predation in larger group sizes. Guppies are frequently found in small groups of two to four individuals under natural conditions, even in high-predation rivers (*41*). Entering an empty arm, either alone or at the front of a group, represents a substantial risk for individuals initiating movement due to the potential presence of undetected predators (*9*, *31*). The design of the maze (Fig. 1A) required the fish to repeatedly make a decision as to which arm to swim into next; thus, as with typical Y-mazes (*9*), the need for decision-making is greatest in the area where the arms meet. Therefore, we focus on the transitions between arms. Behavior within arms (for example, the average swimming speed) is less likely to be in a decision-making context and, thus, more likely to be affected by factors less relevant to decision-making. However, we did find linear relationships between our measures of activity and cohesion and measures of these variables more typically used in experiments of collective movement where animals are not constrained in a maze (fig. S1). A total of 310 fish were each tested once in 102 groups, yielding 4730 movements between the arms of the maze.

### Individual tendencies to initiate and follow

Search, exploration, and activity are major determinants of encountering both resources (*42*) and predators (*39*). Despite decreased activity often being an adaptive response to predation risk (*22*), the total number of movements between arms was not statistically different among fish sympatric with different predators (Fig. 1B and table S2), such that fish from high-predation habitats were not less able or willing to be active and explore their environment. These fish were, however, more homogeneous, with there being less variance in the number of movements between high-predation fish compared to those from medium and low predation habitats (Fig. 1B). This suggests that, at least in a social context, exploration may be under stabilizing selection in habitats where predation is high (*22*).

To further explore increased homogeneity in high-predation habitats, movements between arms were classed as either an initiation (movement into an empty arm) or a follow (movement into an arm occupied by at least one other fish). The time taken for the first fish to follow each initiation was highly positively skewed (fig. S2, A and B), with the majority (73%) of initiations followed within only 2 s. There was no significant difference in the time taken for an initiation to be followed in fish from different predation regimes (table S2). Each initiation was much more likely to be followed by another fish rather than the initiator leaving the arm without being followed, and in trials with four fish, the most frequent outcome for each initiation was for all three potential followers to follow the initiator (fig. S2, C and D). These trends suggest that shoal cohesion was high during the trials and that initiations were frequently successful in leading followers (*43*).

**Fig. 2:**
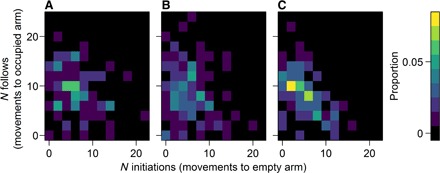
Frequency distribution of the number of initiations versus the number of follows made by each individual fish. Individuals are paneled by the predation risk in their source habitat: low (**A**), medium (**B**), and high (**C**). The color scale indicates the proportion of fish from each level of predation with each combination of the number of initiations and the number of follows, so that the total proportion is equal to 1 in each panel. The negative relationship between initiations and follows is greatest in fish from high-predation habitats, in terms of both the slope of the relationship (negative binomial GLMM predation level × number of initiations: χ^2^ = 5.83, *P* = 0.016) and the correlation (Spearman’s rank correlation: low: *r*_s_ = -0.24, *n* = 84, *P* = 0.026; medium: *r*_s_ = −0.11, *n* = 108, *P* = 0.24; high: *r*_s_ = −0.54, *n* = 118, *P* = 1.78 × 10^−10^).

The number of initiations per fish did not vary with the predation pressure in the fish’s source habitat (fig. S3 and table S2), suggesting that the test arena was not perceived as more or less risky by fish from habitats with different levels of predation. Unlike the total number of movements, the interindividual variability in the number of initiations or follows per fish did not vary with the predation level in the source habitat (fig. S3; negative binomial GLMM on model residuals: initiations: χ^2^ = 0.69, *P* = 0.41; follows: χ^2^ = 0.83, *P* = 0.36). Thus, although the number of initiations and the number of follows are the only components making up the total number of movements, differences in variance in these frequencies cannot explain the homogeneity in the total number of movements observed in fish from high-predation habitats. However, there was a negative relationship between the number of initiations and the number of follows by each fish, suggesting that there may be a trade-off between tendencies to initiate and follow (note that in contrast, individuals could vary in overall activity, generating a positive relationship). This negative relationship was markedly stronger in fish from high-predation habitats (Fig. 2). Together, these results suggest that the greater homogeneity in overall activity (Fig. 1B) in fish from high-predation habitats emerges from the stronger negative correlation between these two behaviors in high-predation fish (Fig. 2C), rather than a reduced variability in initiating and/or following (fig. S3).

### Consistency of initiating over time

The variation between individuals in their tendencies to initiate and follow could be stochastic, without any consistency between individuals even in the short time scale of the trials (*13*). When comparing over time, there was a positive correlation in the number of initiations made by each fish in the first and second half of the trials (fig. S4; Spearman’s rank correlation: low: *r*_s_ = 0.52, *n* = 84, *P* = 3.26 × 10^−7^; medium: *r*_s_ = 0.36, *n* = 108, *P* = 0.00012; high: *r*_s_ = 0.45, *n* = 118, *P* = 3.03 × 10^−7^), demonstrating consistency in the tendency of individuals to initiate movements, at least in the short term. This consistency could be caused by factors such as hunger, which vary over short time scales, or longer-term variation between individuals (*13*, *33*, *44*); retesting individuals over multiple days would be necessary to determine the degree of temporal consistency. There was no evidence that this consistency (the positive relationship in the number of initiations between the two halves of the trials) differed with the level of predation (table S2; negative binomial GLMM predation level × number of initiations in the first half: χ^2^ = 0.28, *P* = 0.60). There was also no difference in the number of initiations made per individual in the first and second half of the trials, suggesting that the fish did not habituate to the experimental arena during the 5-min trials (table S2; negative binomial GLMM: χ^2^ = 1.40, *P* = 0.24).

### Differentiation and feedback within groups

In addition to individual variation at the level of the population, social interactions can result in feedback that acts to magnify (via differentiation) or suppress (via conformity) differences between individuals (*28*, *29*, *45*). One approach to test whether greater skew in decision-making in fish from high-predation habitats was due to differentiation within groups, rather than population-level differences between habitat types, would be to assay tendencies to initiate and follow in isolated individuals and compare this to their behavior in groups [as in the study of Harcourt *et al*. (*28*)]. However, isolation of social animals often changes behavior (*46*), for example, as a result of inducing stress (*47*, *48*), and this effect is likely to vary between our populations as they vary in predation risk, confounding this approach in our study. Thus, we instead conducted two types of randomization simulations based on the data from our experimental trials of fish in groups. In the first randomization, we examined the relationship between the number of initiations made by a fish randomly selected as “fish 1” and another fish randomly selected as “fish 2” from each group (fig. S5). Over multiple groups, a negative slope implies a negative effect of individuals on one another (if one fish in a group initiates a lot, the other does not), whereas a positive slope indicates a positive effect (more initiations by one fish stimulates more initiations by another fish). The random assignment of individuals as fish 1 and fish 2 is iterated 10,000 times to avoid spurious conclusions from any single (entirely arbitrary) assignment of a particular set of fish being labeled fish 1 and fish 2. In fish from low- and medium-predation habitats, there was no evidence of any positive or negative interaction within groups (fig. S5; low, *P* = 0.72; medium, *P* = 0.94). In contrast, there was a statistically significant negative relationship in the number of initiations between fish from high-predation habitats within groups (*P* = 0.004), indicating that there was a negative effect of fish on one another within groups in their tendency to initiate movements into arms.

**Fig. 3:**
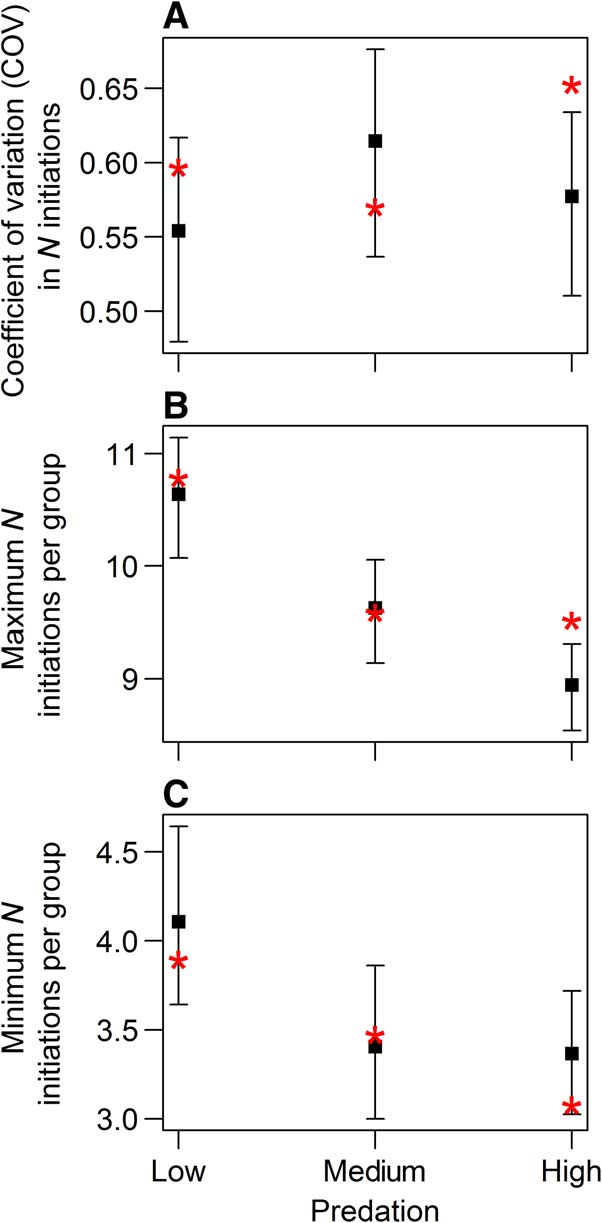
Observed diversity and maximum and minimum number of initiations per group compared to a randomized group membership. The test statistic [COV (**A**), maximum (**B**), and minimum (**C**)] is calculated for each group tested (the mean for each predation level is shown as red asterisks) and plotted against the distribution of the mean of that statistic from 10,000 iterations of a randomization that reforms the groups with random membership within each predation level and group size (due to the significant effect group size on the number of initiations). The means (filled squares) and 95% confidence intervals (error bars) of the randomized distributions are shown. The observed group-level statistic falling outside of the 95% confidence intervals serves as evidence that the composition of the groups in the experiment is significantly different from groups being randomly assembled from the fish tested.

In the second randomization, observed group-level summary statistics were compared to the distribution of these statistics if group membership was randomly shuffled, as has been previously used to test for nonrandom assortment of individuals in groups according to phenotypic traits (*41*, *49*). Unlike the first randomization, the rationale here is to test whether randomly exchanging data of observed behavior for each individual between groups has a significant effect on group level statistics or whether the membership in the observed groups is statistically not important. In each group, we quantified diversity as the coefficient of variation (COV) in the number of initiations between fish in a group. Compared to distributions where individuals were randomly exchanged between groups within a predation level, the observed average diversity was significantly different from expected only in high-predation habitats (Fig. 3A; low, *P* = 0.22; medium, *P* = 0.23; high, *P* = 0.0052), with diversity being higher than expected from chance. This demonstrates that within-group variability in fish from high-predation habitats is dependent on the composition of the groups actually tested and cannot be accounted for by variation across all individuals from high-predation habitats that were tested. Although this suggests behavioral divergence of individuals within groups during the trials, this divergence could be symmetrical (wherein some individuals initiate more than expected and others initiate less) or asymmetrical (wherein either some individuals initiate more or some initiate less than expected) (*50*). Repeating the randomization tests for the individuals that made the most and the fewest initiations in each group suggests that the higher variation than expected by chance in fish from high-predation habitats was primarily due to those with the most initiations increasing their rate of initiation (Fig. 3B; low, *P* = 0.56; medium, *P* = 0.81; high, *P* = 0.0012). This suggests that potential leaders emerged during the trials. There was also a nonsignificant tendency for the individual with the fewest initiations to make fewer initiations than expected (Fig. 3C; low, *P* = 0.37; medium, *P* = 0.70; high, *P* = 0.079). These simulations demonstrate that only in groups from high-predation habitats was the number of initiations made by each fish dependent on the other individuals in the group: Fish differentiated into more “leader” and “follower” types of individuals within groups than would be expected from chance. This differentiation in groups from high-predation habitats can help explain the strong negative relationship between initiating and following in high-predation habitats and, thus, the homogeneity between fish in their total overall activity (Fig. 1B).

### Group cohesion

**Fig. 4:**
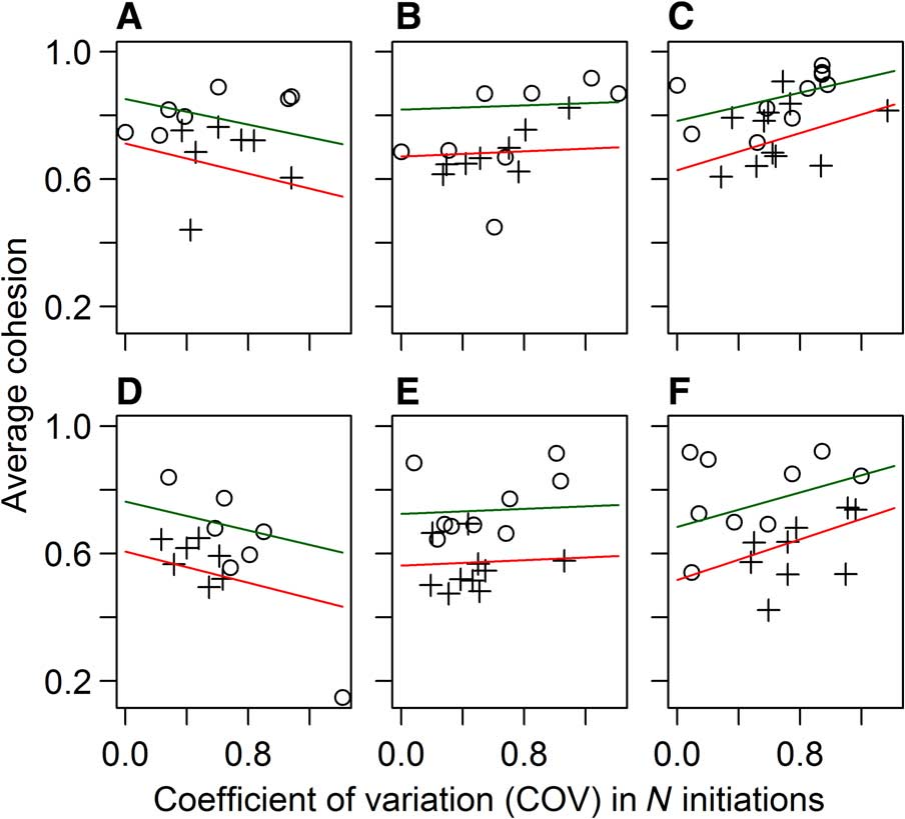
Relationship between diversity in the number of initiations (COV) and group cohesion. Cohesion is calculated as the number of other fish in the same arm/maximum possible number of other fish, averaged over all time frames and across the individuals in a group. Data are paneled by sex [females (**A** to **C**) and males (**D** to **F**)] and predation risk [low (A and D), medium (B and E), and high (C and F)]. Fitted lines are calculated from the GLMM fixed-effect coefficients. Circles and dark green lines represent groups of two fish, and crosses and red lines represent groups of four fish.

To explore group cohesion in a way that was relevant to the shape of the arena and the decisions made to enter arms, we calculated group cohesion for each individual as the number of other fish in the same arm divided by the maximum possible number of other fish in the arm. We averaged this over all time frames per individual and averaged this across the individuals in each group. Although, by definition, groups with a greater COV had a greater skew in which individuals made initiations, group cohesion increased with the COV in fish from high-predation habitats but not in the other, lower-predation habitats (Fig. 4; LMM predation level × COV: *F* = 7.57, *P* = 0.0071). Thus, there may be a functionally important effect of within-group diversity as predation risk generally decreases with group size in a wide range of species (*51*). The differentiation that occurs in fish from high-predation habitats may instead be promoted in more cohesive groups, rather than the differentiation causing greater group cohesion, or alternatively, both of these processes may occur simultaneously. Cohesion and differentiation may both also be affected by a third, unknown variable; for example, in groups of more sociable individuals, both cohesion and differentiation may increase. Further experimental work would be required to determine the mechanism(s) that drives the positive relationship between differentiation and group cohesion. We found no relationship between a group’s COV and the mean number of movements fish made within the group (LMM: COV main effect: *F* = 0.46, *P* = 0.50; predation level × COV: *F* = 0.79, *P* = 0.38), such that more diverse (less egalitarian) groups were not less exploratory, demonstrating that differentiation does not affect, or not affected by, the degree of exploration.

### Effects of sex and group size

Our analyses revealed a number of other significant effects on the response variables that were independent of the predation in the source habitat of the fish (all statistical results are presented in table S2). In larger groups, there were more initiations and follows (and hence, total number of movements); that is, fish were more active per fish. The mean total number of movements between arms per group was greater if there were more initiations on average in the group, as expected, and when groups consisted of females and more individuals (four fish rather than two fish). The number of follows was significantly affected by the interaction between the number of initiations and group size, as the effect of the number of initiations was greater in smaller groups. Groups of females were more cohesive, and this was consistent with females following one another more quickly than males. Larger groups were less cohesive, although cohesion was calculated to be relative to the number of possible fish in an arm, so cohesiveness is a measure relative to total group size.

## DISCUSSION

How influence on group decisions is distributed between individuals (that is, to what extent decisions are equally shared or led by a single individual) is a key question in studying animal collectives. We demonstrate significant variation in collective behavior within a single species over a small geographical range: The furthest two sites were only 18.3 km from one another. This unexpected variation in behavior between collective units has also recently been demonstrated in another well-studied system, the slime mold *Physarum polycephalum* (*52*), where decision-making is distributed between the multiple nuclei occupying each cell. Our study shows significant differences in social behavior between fish from high-predation habitats versus lower-predation habitats; these differences could be direct adaptations to mitigate predation risk or be an indirect consequence of other changes associated with adaptation to predation. Demonstrating a link between decision-making in shoals and survival in ecologically realistic trials with predators would support the hypothesis that changes in decision-making are a direct response to predation pressure. Whatever the underlying mechanism(s) is, our results suggest that it may be difficult to generalize that decision-making within a species is categorically equally shared (that is, egalitarian) or skewed by a small minority (that is, leadership) when based on studies of single populations. Instead, our results suggest group decision-making changes in response to local ecological conditions as do morphological, sensory, and life history traits (*1*).

As our test fish were caught from the wild, differences between fish based on predation in their source habitat could be inherited (either with a genetic basis or via maternal effects) or learned (either through direct experience of predators or indirectly through interacting with more experienced fish). Previous work on guppies has shown a heritable component of shoaling, with the offspring and later descendants of fish originally caught from high-predation habitats showing a stronger shoaling tendency (*20*, *36*). These common garden experiments could be used to determine the extent to which the differences in fish caught from high-predation habitats in the current study are inherited or affected by experience and how persistent different social tendencies are between populations when released from predation in the laboratory. Conversely, how long an exposure to predation is required for initially predator-naïve fish to show the same patterns in group decision-making as fish from high-predation rivers would also be an interesting avenue for future study. There is also evidence that shoaling tendency can be affected by experience. Song *et al*. (*36*) have shown that offspring of fish from high-predation habitats have a stronger shoaling tendency than offspring of fish from low-predation habitats when reared with fish from high-predation, but not low-predation, habitats. If there is a learned component in the differences we observed between fish from habitats varying in predation, this could be demonstrated by exposing predator-naïve fish to predators and testing their collective exploration before and after this exposure. Conducting this experiment with fish with parents from high- or low-predation sites would also reveal whether a flexible response to predation in social behavior is limited to particular populations or is a widespread trend.

It also remains unknown which cues are used by fish from high-predation habitats to differentiate their frequency of initiating movements from others within their groups. It is generally accepted that collective movement in fish shoals is achieved by individuals responding to the positions and movements of their near neighbors, without any active signals contributing to the formation or maintenance of groups (*24*, *53*). Thus, it is feasible that the motion of individuals with weak tendencies to move away from neighbors and into another arm acts to encourage other individuals with a greater tendency to initiate movements, amplifying this tendency. Analyzing fish trajectories to infer the “assertiveness” of individuals (*30*) and how this changes as the trials progress, with respect to the success or failure of previous initiations being followed and the behavior of others in the group, may reveal whether (and if so, which) motion cues are important. However, the number of initiations required to do this would likely be far in excess of the 1884 initiations observed in our study because of the large number of variables that may be important in this analysis.

Given the recent burgeoning of research in animal “personalities” (*45*), it would be of widespread interest to determine whether individuals consistently differ in their propensity to initiate movements over longer time scales, so that particular individuals consistently emerge as those attempting to lead group movements. Our analysis of consistency in the number of initiations per fish was limited only to a short time scale of minutes. The effect of changing individuals between groups [as occurs frequently in animals, such as guppies, that show fission-fusion group dynamics (*41*)] and whether interindividual consistency over longer time periods differs between fish from rivers varying in predation will also help shed light on how influence on decisions in groups is distributed between individuals.

We find that predation is associated with differentiation within groups of guppies, reducing conformity; hence, predation may select against egalitarian, equally shared decision-making. The short duration of our trials (5 min) demonstrates that differentiation can occur very rapidly and over an ecologically relevant time scale (*41*). In the study of Harcourt *et al*. (*28*), which demonstrated the role of social feedback in leadership, pairs of fish interacted for 1 hour, whereas differentiation into specialized “social niches” is generally applied to longer-term, stable social relationships (*54*). In fission-fusion social systems, such as those found in many species of fish-like guppies (*24*), rapid differentiation may allow individuals to quickly establish temporary social roles. Our results also suggest that there may be an interaction between differentiation and cohesion in groups of fish from high-predation sites. If greater differentiation results in higher group cohesion, differentiation is likely to be advantageous in predation avoidance; alternatively, higher group cohesion may cause groups to be more differentiated. The effects of differentiation on the outcomes of group decision-making (for example, the accuracy and speed of decisions and whether this varies with predation pressure) remain to be tested.

## MATERIALS AND METHODS

### Experimental design

The study was designed to track movements of individual fish in shoals as they explored a novel environment. To determine when a decision to move from one area to another occurred, we used a radially symmetric three-armed maze (movie S1). Because of differences in social behavior between males and females (*41*, *55*), only single-sex groups were tested. Under constant laboratory conditions, we tested fish from multiple rivers differing in predation pressure to test for the effect of habitat-level predation on average frequencies and interindividual variation in initiating movements into empty arms and following other fish. To detect whether there was any feedback within groups that resulted in fish initiating more or less than expected by chance, we used randomization tests and ran these separately for fish from each predation regime to determine whether this feedback varied with predation pressure.

### Experimental subjects

Wild adult guppies were caught from six populations, two populations from each of the three rivers in the Northern Range mountains of Trinidad (table S1), using a seine net. Fish were housed and all experimental trials were performed in a laboratory under constant temperature and lighting at the University of West Indies at St. Augustine, Trinidad and Tobago. Fish were held in 300-liter glass tanks of mixed sexes (>100 fish per tank), with only fish caught from a single site kept in each tank, for at least 48 hours before testing. Fish were kept at 23°C throughout and fed ad libitum at the end of each day with tropical flake food. Fish were either returned to their source sites after testing or kept in the laboratory for other behavioral experiments.

### Experimental protocol and materials

Testing took place between 0830 and 1730. The testing arena consisted of a radially symmetric three-armed maze constructed of matt white corrugated plastic (movie S1). Each arm was 36.5 cm long and 10 cm wide, and walls were 18 cm high; the number of initiations into each of the three arms was about equal within each predation level, as were the number of follows (fig. S6). Water depth was 7.5 cm throughout the arena. White plastic sheets surrounded the arena to diffuse overhead fluorescent lighting and minimize disturbance. The arena was filmed from above using a Canon 550D DSLR camera at 25 frames per second and a resolution of 1920 × 1080. A removable door positioned 10 cm from the end of one arm created a start area where the fish were transferred to and habituated in for 2 min before the trial began. The same arm was used for the starting area for all trials. The door was gently lifted at the start of each trial, allowing the fish to explore the arena. Fish were recorded for 5 min, at which point they were removed; each fish was tested only once. Entering an arm was defined as leaving the equilateral triangle at the center of the maze (that is, where the arms meet; the sides of the triangle are 10 cm long). Standard body lengths were measured from the video while the fish were in this central zone, which was directly below the camera. Fish collection and trials were carried out in March and April 2014. All procedures were in accordance with institutional guidelines on animal care and were approved by the University of Bristol Ethical Review Group (UIN/13/028). Data collection was blindly carried out in the sense that the source habitat and sex of fish in each trial were not revealed to the experimenter performing the tracking (C.J.T.).

### Tracking

After starting the video recording, the door was removed, and the white plastic sheets around the arena were closed. The first 30 s of each video was not analyzed because the door being removed obstructed the view of the fish and the lighting in the arena changed from the curtains being opened and then closed. Removing the first 30 s of video ensured that the view of the arena did not change for the remainder of the trial, which facilitated automated tracking, and this also allowed the fish to habituate to any immediate disturbance of removing the door of the starting area (our study was designed to investigate exploration of a novel environment, not fright responses). Fish were then tracked and identified using a machine learning algorithm. The movement of individual fish was recorded throughout each trial, with the fish reidentified following occlusions. This software was implemented in Python, using the OpenCV computer vision library, and followed the approach of idTracker (*56*). Fish were located within images and linked across video frames. When an occlusion occurred between two or more fish, new tracks were created for each individual. The longest segment of tracks without an occlusion was used to train the machine learning classifier, with the features of each individual fish extracted using a rotation-invariant descriptor (*57*). Each subsequent segment was then classified with the sequence of classification determined by the certainty of the identity to track assignment. The certainty was calculated using the confusion matrix of the training sample and the segment that was classified to calculate a likelihood for each permutation of assignments. This uncertainty level was based on the consistency of the identification for each frame of the segment and the similarity between fish, as indicated by the confusion matrix of the training segment. Once a segment was classified, the track assignment and level of certainty were recalculated for each unallocated segment (as tracks often spanned multiple segments, the certainty of all other segments had to be updated). The algorithm proceeded until each segment was identified. The source code for the tracking software is available at https://github.com/ctorney/fishOfInterest.

Fish were allocated to an arm at each frame based on the coordinates (in pixels) of their position. Each arm was defined as the region of the tank beyond the border defined by the equilateral triangle formed at the center of the maze. When in the central region, a fish was considered to still be in the arm it had most recently visited until it crossed the border defined by the edge of the central region and entered a different arm (a transition). By defining transitions in this way, the central region acted as a buffer, and only genuine movements from one arm to another were considered as transitions. To test whether our measures of activity (movements between arms) and cohesion (based on the number of other fish in the same arm as a fish) were related to measures of activity and cohesion often used when individuals are free to move in an open arena, we calculated the average speed and (nearest and mean) neighbor distance using the full trajectory data, that is, including data when fish were not moving between arms. These are commonly used measures of activity and cohesion where individuals are free to move in an open arena (*30*, *58*).

To ensure that misidentification of fish did not play a role in the analysis, the transition data were thresholded at varying levels of the uncertainty measure from the tracking (with 0 being a certain identity assignment and 1 meaning that the algorithm has selected from multiple equally likely identity assignments). Transitions occurring in segments of track that did not meet the threshold certainty level were removed from the analysis. For example, if the threshold uncertainty level was set at 0.05, any time a transition occurred for which the algorithm estimated its likelihood of misidentification to be greater than 0.05, this transition was removed. The effect of applying threshold certainty levels between 0.9 and 0.05 on the number of initiations and number of follows for each fish is shown in fig. S7.

### Statistical analysis

The total number of movements, the number of initiations, and the number of follows made per individual fish were each analyzed as the response variable in mixed models. Models are detailed in table S2. To analyze the degree of interindividual variation between fish from different predation habitats, these models were repeated, with trial identity removed from the random effect. This was removed because the population-level variability around the fitted population value was of interest, controlling for sex and group size effects, not the variance around the fitted value for each trial. The absolute values of the residuals from these models were then analyzed (table S2). To complement this analysis, we compared the variability between individuals from low-, medium-, and high-predation sites using Levene’s tests for homogeneity of variance (*59*), separately for the total number of movements, the number of initiations, and the number of follows per fish. Note that these tests do not take into account any of the other fixed or random terms. Because there were departures from normality in some of these data, medians were used as the center reference values (Levene’s test using the median is also known as the Brown-Forsythe test).

The (log_10_-transformed) time taken for each initiation to be followed by the first fish was also analyzed. Initiations that were not followed [that is, the initiating fish left the arm before another fish entered that arm; 406 of 1884 initiations (21.5%)] were excluded from this analysis. To explore the relationship between the number of initiations and follows for each fish, the number of follows was analyzed as a function of the number of initiations. The number of initiations per fish in the second half of the trials was analyzed as a function of the number of initiations in the first half of the trials to test for consistency in tendencies to initiate within the duration of the trials. The number of initiations per fish was also compared between the first and second halves of the trials to test whether more initiations took place as the trials progressed, which would be suggestive of habituation to the test arena (table S2).

Mean group cohesion was arcsine square root–transformed before being analyzed using an LMM. Predation level, sex, and group size were included as explanatory variables, as well as the mean and the COV, in the number of initiations of fish in a trial. Because there were five main effects and a smaller sample size compared to other analyses, only the interaction between predation level and the COV was included in the analysis. River was included as a random effect. This analysis was repeated for the mean number of total movements (both initiations and follows) as a response variable (table S2).

Fish body size was included as an additional main effect throughout; because this covaried with sex, it was normalized using the Student’s *t* statistic within each sex. Nonsignificant interactions were removed (table S2). Analyses assuming a normal distribution were checked for model assumptions using diagnostic plots (homogeneity of variance and normality in residuals), whereas those assuming a negative binomial or binomial distribution were checked for the dispersion parameter being approximately 1 (that is, >0.5 and <2). All tests were two-tailed. All analyses were carried out in R version 3.0.2 (*60*).

## Supplementary Material

http://advances.sciencemag.org/cgi/content/full/3/5/e1602682/DC1

## SUPPLEMENTARY MATERIALS

Supplementary material for this article is available at http://advances.sciencemag.org/cgi/content/full/3/5/e1602682/DC1 fig. S1. Relationships between measures of activity and cohesion in this study versus those more typically used, wherein animals are unconstrained by a maze. fig. S2. Following behavior in the trials. fig. S3. The effect of predation risk in the source habitat on the number of initiations and number of follows per individual fish. fig. S4. Relationship between the number of initiations made by each fish in the first versus second half of each trial. fig. S5. Relationship between the number of initiations made by two fish in groups from each predation level. fig. S6. Frequency of using each arm for initiations and follows by fish from habitats with different levels of predation risk. fig. S7. Effect of applying a threshold based on the level of uncertainty of whether a segment of track belongs to a particular fish. table S1. Populations, their level of predation, and the location where they were sampled. table S2. Full statistical results for linear models including sample sizes. table S3. Summary of sample sizes. movie S1. Example of group decisions and collective movement of guppies in the three-armed maze. data file S1. Data for individual fish behavior, aggregated over each trial. data file S2. Data for individual fish behavior, aggregated over the first and second halves of the trials.
