# Supplementary material for: High-predation habitats affect the social dynamics of collective exploration in a shoaling fish
Source: Sci Adv. 2017 May 3;3(5):e1602682. doi: 10.1126/sciadv.1602682 (PMC5415332; doi:10.1126/sciadv.1602682)
Supplement: http://advances.sciencemag.org/cgi/content/full/3/5/e1602682/DC1 [file supp_3_5_e1602682__index.html]

Science Advances | Science Advances

## Supplementary Materials

**This PDF file includes:**

- fig. S1. Relationships between measures of activity and cohesion in this study versus those more typically used, wherein animals are unconstrained by a maze.
- fig. S2. Following behavior in the trials.
- fig. S3. The effect of predation risk in the source habitat on the number of initiations and number of follows per individual fish.
- fig. S4. Relationship between the number of initiations made by each fish in the first versus second half of each trial.
- fig. S5. Relationship between the number of initiations made by two fish in groups from each predation level.
- fig. S6. Frequency of using each arm for initiations and follows by fish from habitats with different levels of predation risk.
- fig. S7. Effect of applying a threshold based on the level of uncertainty of whether a segment of track belongs to a particular fish.
- table S1. Populations, their level of predation, and the location where they were sampled.
- table S2. Full statistical results for linear models including sample sizes.
- table S3. Summary of sample sizes.
- Legend for movie S1
- Legends for data files S1 and S2

Download PDF

**Other Supplementary Material for this manuscript includes the following:**

- movie S1 (.mov format). Example of group decisions and collective movement of guppies in the three-armed maze.
- data file S1 (Microsoft Excel format). Data for individual fish behavior, aggregated over each trial.
- data file S2 (Microsoft Excel format). Data for individual fish behavior, aggregated over the first and second halves of the trials.

**Files in this Data Supplement:**

- Adobe PDF - 1602682\_SM.pdf
