# Supplementary material for: High-predation habitats affect the social dynamics of collective exploration in a shoaling fish
Source: Sci Adv. 2017 May 3;3(5):e1602682. doi: 10.1126/sciadv.1602682 (PMC5415332; doi:10.1126/sciadv.1602682)
Supplement: http://advances.sciencemag.org/cgi/content/full/3/5/e1602682/DC1 [file 1602682_SM.pdf]

Science Advances

AAAS

advances.sciencemag.org/cgi/content/full/3/5/e1602682/DC1

Supplementary Materials for

High-predation habitats affect the social dynamics of collective exploration in a shoaling fish

Christos C. Ioannou, Indar W. Ramnarine, Colin J. Torney

Published 3 May 2017, *Sci. Adv.* **3**, e1602682 (2017)
DOI: 10.1126/sciadv.1602682

The PDF file includes:

- fig. S1. Relationships between measures of activity and cohesion in this study versus those more typically used, wherein animals are unconstrained by a maze.
- fig. S2. Following behavior in the trials.
- fig. S3. The effect of predation risk in the source habitat on the number of initiations and number of follows per individual fish.
- fig. S4. Relationship between the number of initiations made by each fish in the first versus second half of each trial.
- fig. S5. Relationship between the number of initiations made by two fish in groups from each predation level.
- fig. S6. Frequency of using each arm for initiations and follows by fish from habitats with different levels of predation risk.
- fig. S7. Effect of applying a threshold based on the level of uncertainty of whether a segment of track belongs to a particular fish.
- table S1. Populations, their level of predation, and the location where they were sampled.
- table S2. Full statistical results for linear models including sample sizes.
- table S3. Summary of sample sizes.
- Legend for movie S1
- Legends for data files S1 and S2

Other Supplementary Material for this manuscript includes the following:
(available at advances.sciencemag.org/cgi/content/full/3/5/e1602682/DC1)

- movie S1 (.mov format). Example of group decisions and collective movement of guppies in the three-armed maze.

- data file S1 (Microsoft Excel format). Data for individual fish behavior, aggregated over each trial.
- data file S2 (Microsoft Excel format). Data for individual fish behavior, aggregated over the first and second halves of the trials.

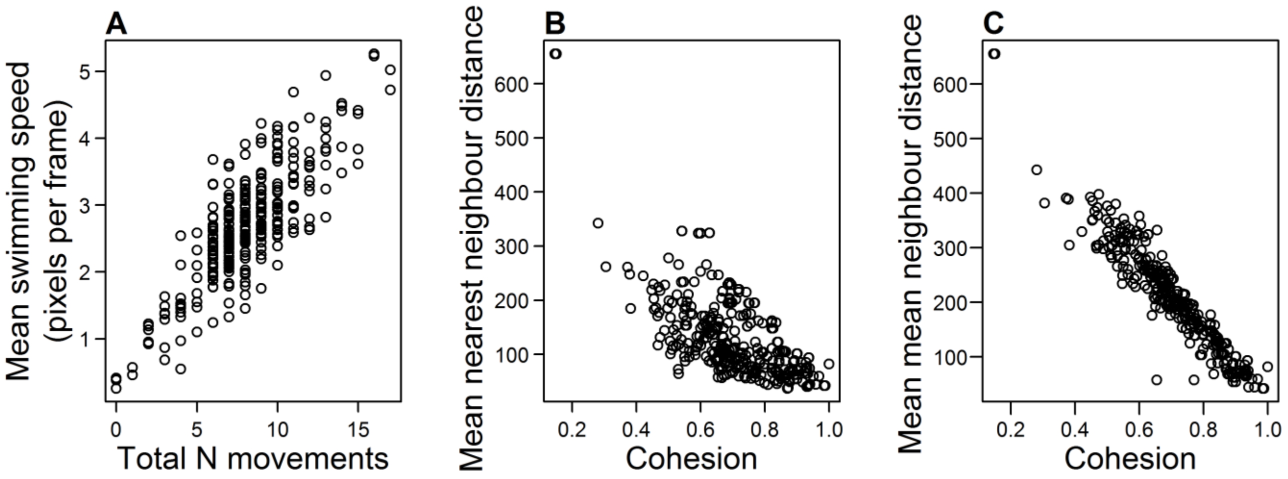

fig. S1. Relationships between measures of activity and cohesion in this study versus those more typically used, wherein animals are unconstrained by a maze. Our study uses the number of transitions between arms per fish as a measure of their activity; this correlates positively with the average swimming speed of each fish (**A**: Spearman's rank correlation: r_s = 0.75, n = 310, $P < 2.2\times10^{-16}$). The cohesion measure for each fish, measured in our study as the number of other fish in the same arm / maximum possible number of other fish, averaged over all time frames, is negatively correlated with the nearest (**B**: Spearman's rank correlation: r_s = -0.65, n = 310, $P < 2.2\times10^{-16}$) and mean (**C**: Spearman's rank correlation: r_s = -0.95, n = 310, $P < 2.2\times10^{-16}$) neighbour distances for each fish averaged over all time frames. Note that the correlation between the two measures of cohesion in C are more strongly correlated than in B as both measures are 'global' measures of cohesion which take into account all other group members, while in B, the nearest neighbour distance is a more 'local' measure that only considers the nearest fish to the focal individual. Average swimming speed and neighbour distances are calculated from the fish's tracking data throughout the trial, not just when they are moving between arms.

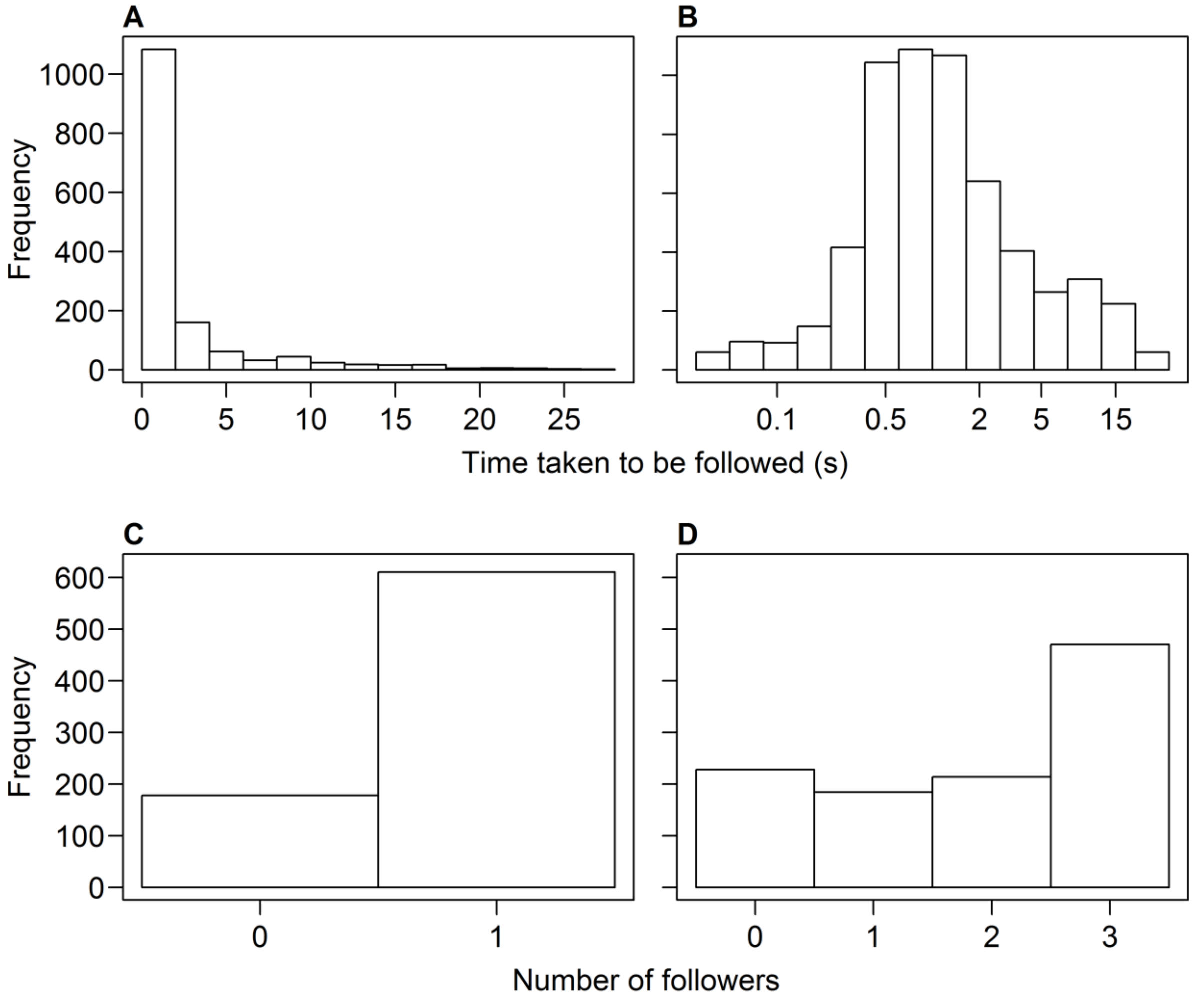

fig. S2. Following behavior in the trials. When initiations were followed, the first fish to follow typically did so within a few seconds (**A**, **B**). The time taken is shown on both linear (A) and log10 (B) scales, with initiations that are not followed omitted. In trials with 2 (**C**) and 4 (**D**) fish, the most common outcome by far after an initiation was for all the other fish in the group to enter the same arm as the initiator, before the initiator later left that arm.

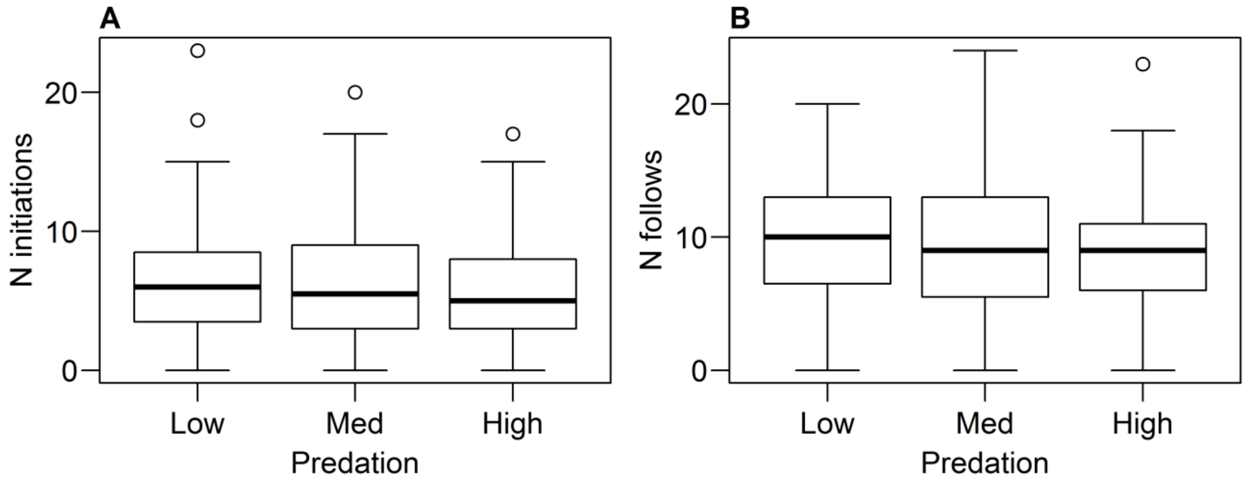

fig. S3. The effect of predation risk in the source habitat on the number of initiations and number of follows per individual fish. Shown are the number of initiations (**A**) and number of follows (**B**). The median is shown by the solid line, the inter-quartile range is enclosed by the box, the whiskers extend to the most extreme data point within 1.5 × the interquartile range outside the box, and empty circles show data points beyond the range of the whiskers. There was no difference between fish from different predation regimes in either their average number of initiations or follows (table S2) or in the variability between fish in these frequencies (table S2; Levene's test for homogeneity of variance: number of initiations: $F_{2,307} = 0.36$, $P = 0.70$, number of follows: $F_{2,307} = 2.39$, $P = 0.094$).

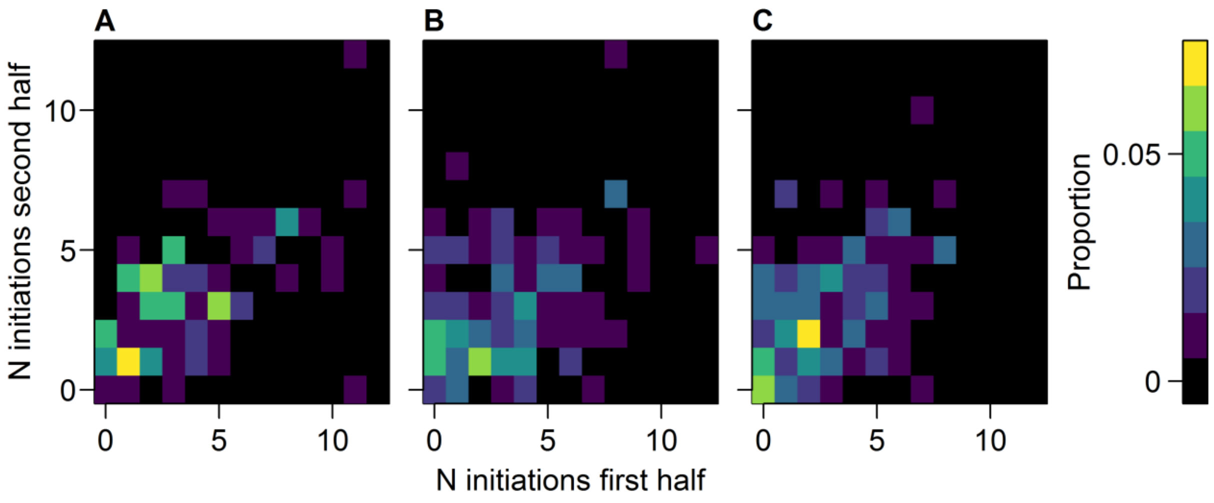

fig. S4. Relationship between the number of initiations made by each fish in the first versus second half of each trial. Individuals are paneled by the predation risk in their source habitat: (**A**) low, (**B**) medium and (**C**) high. The color scale indicates the proportion of fish from each level of predation with each combination of number of initiations and number of follows, so that the total proportion is equal to 1 in each panel. Fish from all habitat types demonstrated consistent tendencies to initiate movements into empty arms over the time scale of the trials.

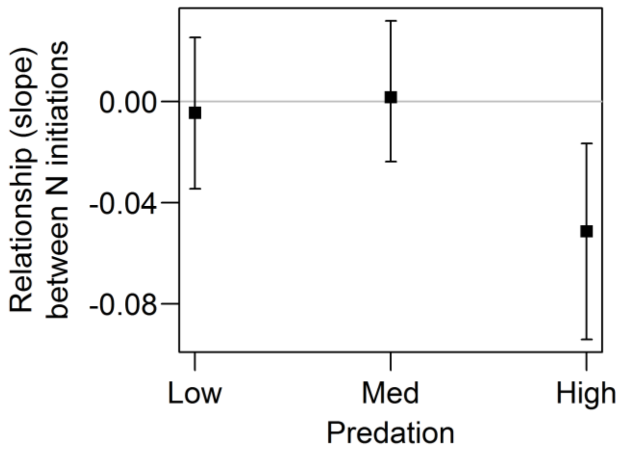

fig. S5. Relationship between the number of initiations made by two fish in groups from each predation level. Two fish were randomly selected (without replacement) in each group as 'fish 1' and 'fish 2'. Over all groups in each predation level (low, medium and high), the relationship between the number of initiations made by fish 2 as a function of the number of initiations made by fish 1 was determined by the gradient of the slope from a negative binomial GLM (with sex and group size included as main effects; data were analysed separately for each predation level). This randomisation was iterated 10,000 times to generate the mean (filled squares) and 95% confidence intervals (error bars) of the relationship in number of initiations between pairs of fish. Values greater than 0 indicate a positive effect of one individual on the other, 0 suggests no effect, and values less than 0 indicate a negative effect within groups. Only in groups from high predation habitats is there evidence of interactions between individuals, as the 95% confidence intervals of the distribution do not overlap with 0.

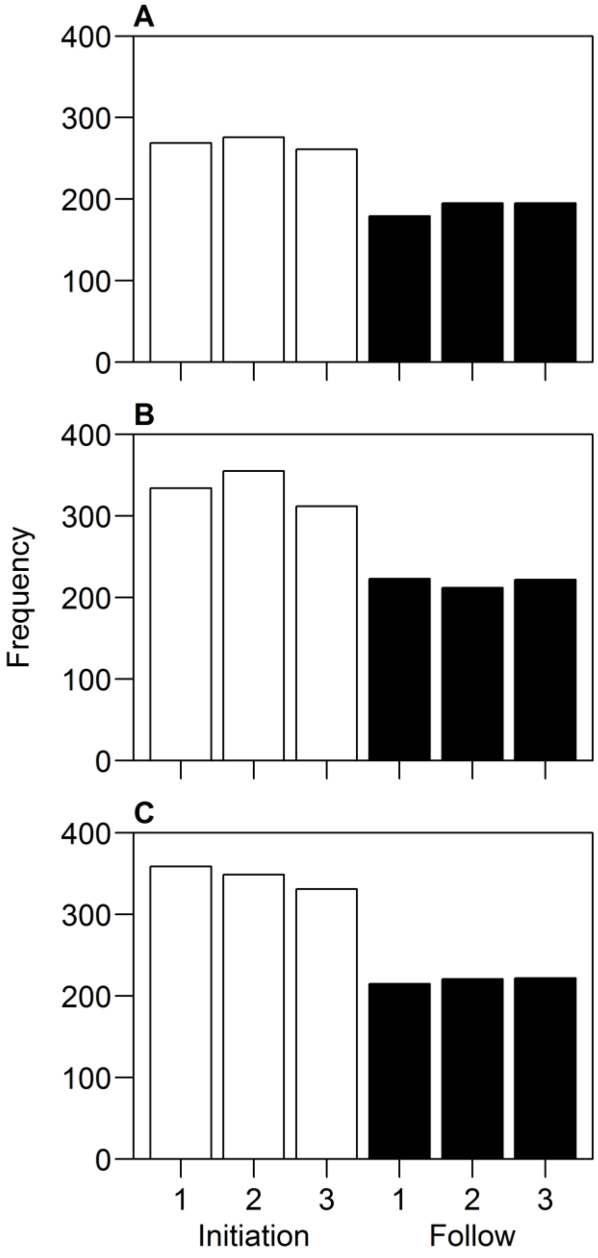

fig. S6. Frequency of using each arm for initiations and follows by fish from habitats with different levels of predation risk. Panels show trials of groups from low (**A**), medium (**B**) and high (**C**) predation risk habitats. Empty bars are movements into the arm when it is empty (i.e. initiations) and filled bars are movements into the arm when it is already occupied by at least one other fish (i.e. follows). In the x axis, the three arms are labelled as 1, 2 and 3.

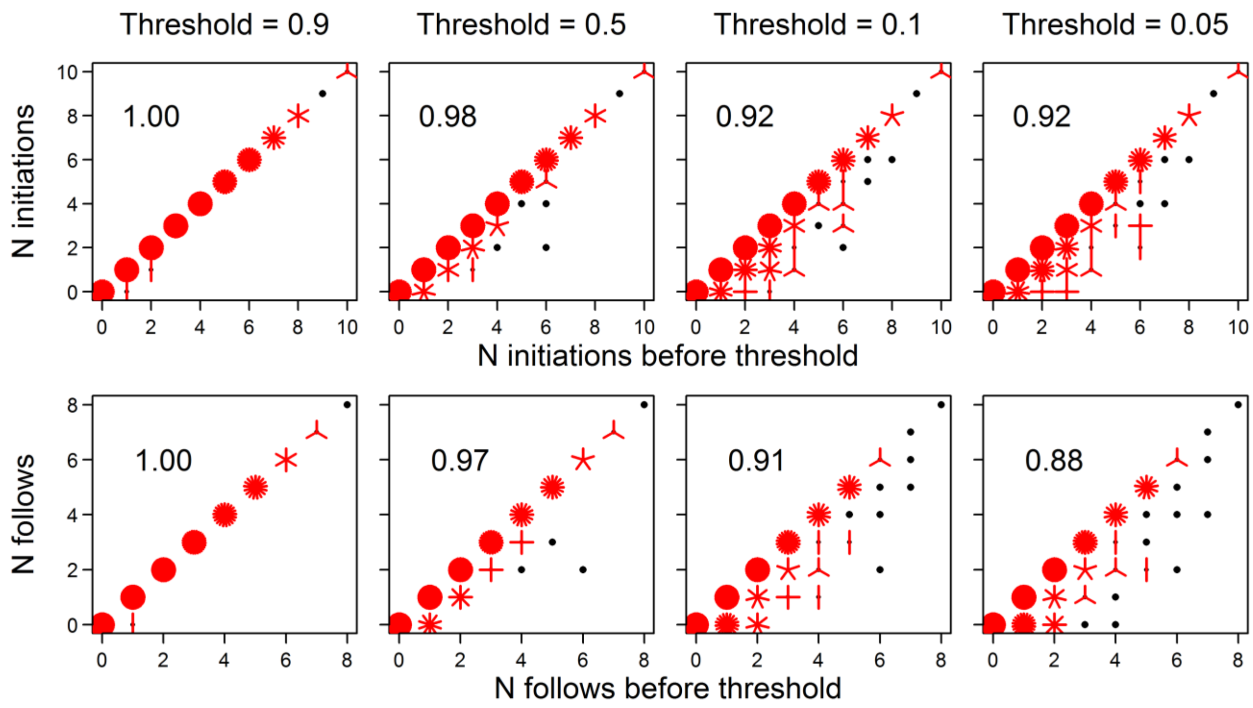

fig. S7. Effect of applying a threshold based on the level of uncertainty of whether a segment of track belongs to a particular fish. The number of initiations (top row) and number of follows (bottom row) of each fish before and after the threshold is applied is plotted in each panel. The threshold level of uncertainty before segments of track are excluded decreases from the left-most to right-most column (i.e. the threshold becomes more stringent). An uncertainty level of 0 is a certain assignment of identity, and 1 means the algorithm has selected from multiple equally likely identity assignments. The value in the top left of each panel shows the Spearman's rank correlation coefficient of the relationship in each panel, rounded to two decimal places. Overlapping cases are represented by multiple red lines radiating from that coordinate, with one line per overlapping case.

table S1. Populations, their level of predation, and the location where they were sampled.

Population	Predation	Longitude	Latitude
Lower Lopinot	High	10:41.7	-61:19.26
Upper Lopinot	Medium	10:43.0	-61:19.28
Lower Aripo	High	10:39.1.3	-61:13.24.6
Upper Aripo	Low	10:40.46.9	-61:13.42.8
Lower Turure	Medium	10:39.24.2	-61:10.4.9
Upper Turure	Low	10:41.2	-61:10.30

table S2. Full statistical results for linear models including sample sizes. LMM refers to Linear Mixed Model, GLMM to Generalised Linear Mixed Model and Neg. bin. to negative binomial. Unless otherwise stated, the 1st Step of a model includes all two-way interactions (with normalised body size (Norm_size) only as a main effect) and the 2nd Step to the simplified model with non-significant two-way interactions removed. River is Aripo, Lopinot or Turure. Norm_size is the size of the fish normalised within each sex, and N_initiations refers to the number of initiations made by a fish over a trial. Rank refers to an individual's rank within their group based on the number of initiations made. Half refers to whether initiations were made in the first or second half of each trial. Full colons denote interaction terms. The Statistic refers to either the F test statistic (for LMMs) or the Chisq test statistic (for GLMMs). Effects with $P < 0.05$ are indicated in bold font.

Response variable and sample size	Test	Random effect	Step	Fixed effect	Statistic	P value
Total number of movements between arms (i.e. activity) (N = 310)	LMM	River / Trial	1st	Predation:Sex	1.80	0.18
				Predation:Group_size	0.066	0.80
				Sex:Group_size	0.40	0.53
			2nd	Predation	0.27	0.61
				Sex	0.31	0.58
				**Group_size**	**5.76**	**0.018**
				Norm_size	0.13	0.72
Absolute residuals of N movements (N = 310)	Neg. bin. GLMM	River / Trial	NA	**Predation**	**6.70**	**0.0098**
				Group_size	0.21	0.65
				Sex	0.52	0.47
				Norm_size	0.49	0.48
Time taken for each initiation to be followed (N = 1478)	LMM	River / Trial / Fish	1st	Predation:Sex	2.17	0.14
				Predation:Group_size	2.89	0.09
				Sex:Group_size	0.26	0.61
			2nd	Predation	1.10	0.31
				Sex	**20.27**	**1.98×10^{-5}**
				Group_size	1.97	0.16
				Norm_size	0.16	0.69
Number of initiations (N = 310)	Neg. bin. GLMM	River / Trial	1st	Predation:Sex	0.23	0.63
				Predation:Group_size	0.066	0.80
				Sex:Group_size	0.012	0.91
			2nd	Predation	1.28	0.26
				Sex	0.37	0.54
				**Group_size**	**24.68**	**6.76×10^{-7}**
				Norm_size	0.048	0.83
Absolute residuals of N initiations (N = 310)	Neg. bin. GLMM	River / Trial	NA	Predation	0.69	0.41
				Group_size	0.96	0.33
				Sex	0.92	0.34
				Norm_size	0.0007	0.98
Number of follows (N = 310)	Neg. bin. GLMM	River / Trial	1st	Predation:Sex	1.01	0.32
				Predation:Group_size	0.075	0.78
				Sex:Group_size	0.18	0.68
			2nd	Predation	0.37	0.55
				Sex	1.82	0.18
				**Group_size**	**73.87**	**$<2\times10^{-16}$**
				Norm_size	0.35	0.55
Absolute residuals of N follows (N = 310)	Neg. bin. GLMM	River / Trial	NA	Predation	0.83	0.36
				Group_size	0.65	0.42
				Sex	0.0040	0.95
				Norm_size	0.58	0.45

Number of follows (N = 310)	Neg. bin. GLMM	River / Trial	1st	Predation:Sex	3.05	0.081
				**N_initiations:Group_size**	**4.57**	**0.032**
				Sex:N_initiations	0.70	0.40
				**N_initiations:Predation**	**6.71**	**0.0096**
				Sex:Group_size	0.26	0.61
				Predation:Group_size	0.20	0.65
			2nd	Sex	0.23	0.63
				Norm_size	0.11	0.74
				**N_initiations:Predation**	**5.83**	**0.016**
				**N_initiations:Group_size**	**3.95**	**0.047**
Number of initiations in second half of trials (N = 310)	Neg. bin. GLMM	River / Trial	1st	Predation:Sex	0.098	0.75
				N_initiations 1st half:Group_size	1.00	0.32
				Sex:N_initiations 1st half	2.10	0.15
				N_initiations 1st half:Predation	0.28	0.60
				Sex:Group_size	0.73	0.39
				Predation:Group_size	0.0077	0.93
			2nd	Predation	3.24	0.072
				Sex	1.016	0.31
				Norm_size	0.76	0.38
				**N_initiations 1st half**	**64.35**	**1.043×10^{-15}**
				**Group_size**	**29.56**	**5.42×10^{-8}**
Number of initiations (N = 620)	Neg. bin. GLMM	River / Trial / Fish	1st	Predation:Sex	0.26	0.61
				Predation:Group_size	0.22	0.64
				Sex:Group_size	0.0003	0.99
				Predation:Half	1.53	0.22
				Sex:Half	0.0007	0.98
				Group_size:Half	0.010	0.92
			2nd	Predation	1.17	0.28
				Half	1.40	0.24
				Sex	0.47	0.49
				**Group_size**	**24.08**	**9.27×10^{-7}**
				Norm_size	0.00	1.00

Mean cohesion (N = 102)	LMM	River	NA	**Sex**	**19.42**	**2.80×10^{-5}**
				**Group_size**	**32.47**	**1.42×10^{-7}**
				Mean_N_initiations	0.51	0.48
				Mean_norm_size	0.13	0.72
				**COV:Predation**	**7.57**	**0.0071**
Mean total number of movements between arms (N = 102)	LMM	River	1st	**Sex**	**8.17**	**0.0052**
				**Group_size**	**294.0**	**$<2\times10^{-16}$**
				**Mean_N_initiations**	**553.0**	**$<2\times10^{-16}$**
				Mean_norm_size	1.08	0.30
				COV:Predation	0.79	0.38
			2nd	**Sex**	**8.07**	**0.0055**
				**Group_size**	**301.1**	**$<2\times10^{-16}$**
				**Mean_N_initiations**	**557.5**	**$<2\times10^{-16}$**
				Mean_norm_size	0.75	0.39
				COV	0.46	0.50
				Predation	0.12	0.73

table S3. Summary of sample sizes. The number of fish and groups are split by group size, sex and population. The predation level of each population is also given.

Group size 2							
Population	Predation	N fish			N groups		
		Male	Female	Total	Male	Female	Total
Lower lopinot	High	10	8	18	5	4	9
Upper lopinot	Medium	8	6	14	4	3	7
Lower aripo	High	8	12	20	4	6	10
Upper aripo	Low	6	8	14	3	4	7
Lower turure	Medium	10	12	22	5	6	11
Upper turure	Low	8	6	14	4	3	7
Group size 4							
Population	Predation	N fish			N groups		
		Male	Female	Total	Male	Female	Total
Lower lopinot	High	16	20	36	4	5	9
Upper lopinot	Medium	16	16	32	4	4	8
Lower aripo	High	20	24	44	5	6	11
Upper aripo	Low	12	16	28	3	4	7
Lower turure	Medium	24	16	40	6	4	10
Upper turure	Low	16	12	28	4	3	7

movie S1. Example of group decisions and collective movement of guppies in the three-armed maze. Superimposed on each fish is a coloured box which indicates each individual's identity that is tracked throughout the trial. The fish are females from the Lower Lopinot, a high predation habitat.

data file S1. Data for individual fish behavior, aggregated over each trial. Predation is 0 (low), 1 (medium) or 2 (high), and Sex 0 (male) or female (1). N_empty is the number of movements into an empty arm, i.e. initiations, and N_transitions to the total number of movements. N_occupied refers to the number of follows, i.e. movements into arms already occupied by other fish. Size is the body length of each fish in pixels. Norm_size is the size of the fish normalised within each sex. Av_neighbours is the number of other fish in an arm / maximum possible number of other fish, averaged over all time frames.

data file S2. Data for individual fish behavior, aggregated over the first and second halves of the trials. Columns headers in common with data S1 have the same meanings and coding. N_movements is the number of movements that are initiations (when Empty arm? = 1) and follows (when Empty arm? = 0).
